# Strategy for the Conservative Treatment of Type-III Camptodactyly in Children with Beals-Hecht Syndrome

**DOI:** 10.1055/s-0041-1739401

**Published:** 2021-12-06

**Authors:** Maria da Conceição Soares de Oliveira, Saulo Fontes Almeida, Anderson Vieira Monteiro, Maria Caroliny Soares de Oliveira, Felipe Soares Figueiredo, Diego Pinheiro Aguiar

**Affiliations:** 1 Área de Reabilitação, Instituto Nacional de Traumatologia e Ortopedia, Rio de Janeiro, RJ, Brasil; 2 Centro de Cirurgia da Mão, Instituto Nacional de Traumatologia e Ortopedia, Rio de Janeiro, RJ, Brasil; 3 Divisão de Pesquisa, Instituto Nacional de Traumatologia e Ortopedia, Rio de Janeiro, RJ, Brasil; 4 Laboratório de Biomodelos e Prototipagem, Centro Universitário Estadual da Zona Oeste, Rio de Janeiro, RJ, Brasil

**Keywords:** congenital hand deformities, finger joints, orthoses, rehabilitation

## Abstract

The authors present a successful case in the conservative treatment of type-III camptodactyly in a patient with Beals-Hecht syndrome. Camptodactyly is a flexion deformity of the proximal interphalangeal (PIP) joint, in the anteroposterior direction, painless and bilateral in 2/3 of the cases. Type-III is the most severe and disabling form, as it usually affects several fingers and is associated with syndromes and other malformations. The case herein reported had the correction achieved with the systematic use of static orthoses started at 7 months of age and completed after 23 and a half months of the intervention.

## Introduction

Camptodactyly is a flexion deformity of the proximal interphalangeal (PIP) joint of congenital and non-traumatic origin. It is a rare condition, with a prevalence of approximately 1%, with the fifth finger being the most affected. 1 It is classified into types I, II and III. Type I: infantile camptodactyily, which usually affects the little finger in isolation. Type II: adolescent camptodactyl, which has a rapid evolution with the growth spurt. Type III: present at birth, it affects several fingers, and is associated with other syndromes. Camptodactyly affects structures that cross the joint, and they are implicated as one of the possible causes. Changes in the skin, aponeurosis, tendons, lumbrical muscle, superficial flexor muscle of the fingers and ligaments can be found. 1 The initial treatment is conservative, with the use of orthoses and passive stretching, or surgical stretching. 2 In cases in which flexion of the PIP joint is ≥ 60°, surgery is the choice procedure. 3 4

Beals-Hecht syndrome is a rare syndrome diagnosed in less than 1 in 10 thousand patients worldwide. 5 6 The clinical picture consists of congenital contractures of multiple joints, 7 long and slender limbs, congenital contratural arachnodactyly, kyphoscoliosis, and anomalies of the auricular pavilions. 3 The contractures may reduce in severity, but the camptodactyly present in the fingers persists. 7

## Case Report

**Fig. 1:**
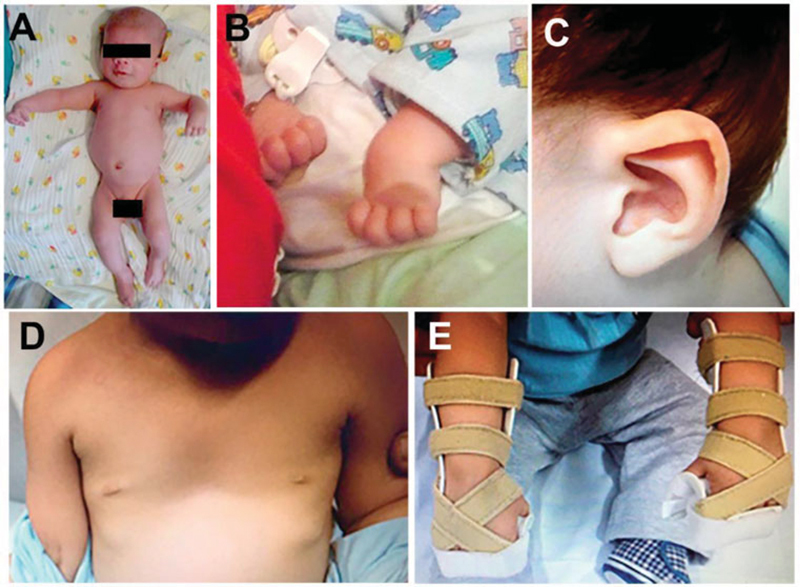
Phenotypic features of the patient with Beals-Heacht syndrome. Two-month-old patient ( **A** ) with deformities in the wrist and metacarpophalangeal and proximal interphalangeal joints ( **B** ), anomalies in the auricular pavilion ( **C** ), pectus excavatum ( **D** ), and positioning orthosis with dorsal support ( **E** ).

A 6-month-old white male patient cared for at the Hand Surgery Outpatient Clinic of our institution for the first time in October 2013. The presence of some clinical signs was observed: PIP joint flexion of the middle, ring and little fingers, flexion of the wrists and hyperextension of the metacarphalangeal (MCP) joints bilaterally, changes in the auricular pavilion, and pectus excavatum. All the contractures present were rigid. He was diagnosed with type-III camptodactyily, and we opted for the conservative treatment with orthotization in November 2013. The initial goniometry of the patient was not performed due to the difficulty in execution, and a photographic record was made ( Fig. 1A-E ). The flexion contractures of the PIP joint presented more than 90°. The use of static orthoses was initially uninterrupted, 8 and the follow-up visits occurred every three months for adjustments in the orthoses or to change them.

**Fig. 2:**
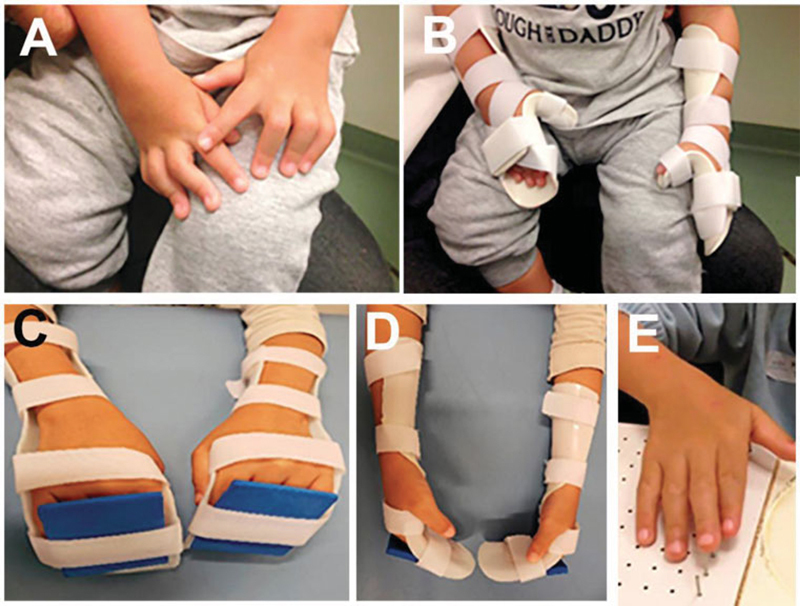
Demonstration of the evolution of the conservative treatment of the patient with camptodactyly. Beginning of the correction ( **A** ), positioning orthosis with maintenance of the dorsal support ( **B** ), nocturnal orthosis with free thumbs and ethylene-vinyl acetate (EVA) plate in the dorsal region of the fingers ( **C** ), nightly use of orthosis in lateral view ( **D** ) and complete extension of fingers ( **E** ).

The orthotization process began at seven months of age. The initial objective was to reach the neutral position of the PIP joint flexion, wrist flexion and MCP joint hyperextension, achieved in February 2015 ( Fig. 2 A-E ).

After reaching the neutral position, the orthosis was modified, starting with a slight flexion of the MCP joint and dorsal support device following the angulation of the orthosis to exert pressure on the fingers against the orthosis, thus maintaining the position besides assisting the flexion of the MCP. From July 2015, already at 26 months, the orthosis began to be made with free thumbs, flexed MCP joint, and wrist in extension ( Fig. 2, A-D ).

**Fig. 3:**
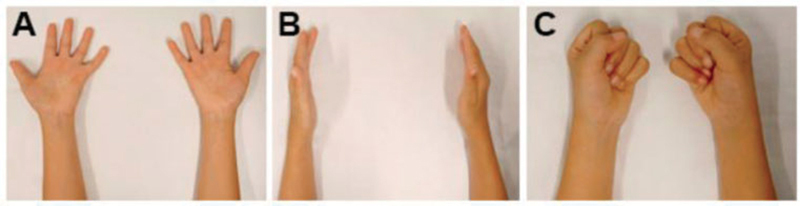
Morphology of the upper limb after conservative treatment for 23 and a half months. Supine position with the fingers in extension ( **A** ), side view ( **B** ), fingers in flexion ( **C** ).

The orthoses started to be used only at night, around 8 p.m., since June 2016. Correction of the camptodactyly was achieved ( Fig. 2E and Fig. 3A-C) .

## Discussion

In this case of rigid type-III camptodactyly in children with Beals-Heacht syndrome, early intervention 9 with static orthoses provided a satisfactory outcome with the regression of the deformity and the achievement of functional use of the hands. The use of orthoses should be the first choice for the beginning of the treatment, even syndromic cases. 9

The multiplicity of structures involved in camptodactyly that are responsible for joint imbalance can reach balance with tissue remodeling using orthoses. 9

The use of orthoses to remodel musculoskeletal tissue is a low-complexity option, but there is a need for a relatively long period of use for the remodeling to occur. 10

The use of the dorsal support device optimized the elongation of the structures responsible for flexion contracture in the PIP joint.

Clinical follow-up of the patient in question will be maintained until the end of the skeletal growth to avoid possible recurrence of soft-tissue contractures. 4 8

The involvement of the family and their collaboration made a difference in the conservative treatment. The intervention is relatively long and exhaustive, but necessary. 10
