# Supplementary material for: Strategy for the Conservative Treatment of Type-III Camptodactyly in Children with Beals-Hecht Syndrome
Source: Rev Bras Ortop (Sao Paulo). 2021 Dec 6;59(3):e485–8. doi: 10.1055/s-0041-1739401 (PMC11193586; doi:10.1055/s-0041-1739401)
Supplement: Related texts

# Research article: Estratégia para o tratamento conservador da camptodactilia de tipo III em criança com síndrome de Beals-Hecht

**Keywords:** articulações dos dedos, deformidades congênitas da mão, órteses, reabilitação

## Abstract

Os autores apresentam um caso bem-sucedido no tratamento conservador da camptodactilia de tipo III em paciente com síndrome de Beals-Hecht. A camptodactilia é uma deformidade em flexão da articulação interfalangeana proximal (IFP), no sentido anteroposterior, indolor, e bilateral em 2/3 dos casos. A de tipo III é a forma mais grave e incapacitante, pois geralmente acomete vários dedos e está associada a síndromes e outras malformações. O caso apresentado teve a correção alcançada com o uso sistemático de órteses estáticas iniciado aos 7 meses de idade e concluído após 23 meses e meio de intervenção.

## Introdução

A camptodactilia é uma deformidade em flexão da articulação interfalangeana proximal (IFP), de origem congênita e não traumática. É uma condição rara, com prevalência em torno de 1%, sendo o quinto dedo o mais afetado. 1 É classificada em tipos I, II e III. Tipo I: camptodactilia infantil, que acomete geralmente o dedo mínimo de forma isolada. Tipo II: camptodactilia da adolescência, de rápida evolução com o estirão do crescimento. Tipo III: presente ao nascimento, acomete vários dedos, e está associada a outras síndromes. A camptodactilia acomete estruturas que cruzam a articulação, e elas estão implicadas como uma das possíveis causas. Podem ser encontradas alterações na pele, aponeurose, tendões, músculo lumbrical, músculo flexor superficial dos dedos, e ligamentos. 1 O tratamento inicial é o conservador, com o uso de órteses e alongamentos passivos, ou o cirúrgico. 2 Em casos em que a flexão da articulação IFP é ≥ a 60°, a indicação é cirúrgica. 3 4

A síndrome de Beals-Hecht é uma síndrome rara, diagnosticada em menos de 1 a cada 10 mil pacientes no mundo. 5 6 O quadro clínico consiste em contraturas congênitas de múltiplas articulações, 7 membros longos e delgados, aracnodactilia contratural congênita, cifoescoliose, e anomalias dos pavilhões auriculares. 3 As contraturas podem reduzir de gravidade, mas a camptodactilia presente nos dedos persiste. 7

## Relato de Caso

Paciente masculino, de 6 meses de idade, branco, atendido no Ambulatório de Cirurgia da Mão de nossa instituição pela primeira vez em outubro de 2013. Observou-se a presença de alguns sinais clínicos, a saber: flexão das articulações IFPs dos dedos médio, anelar e mínimo, flexão de punhos, e hiperextensão das articulações metacarpofalangeanas (MCFs) bilateralmente, alterações em pavilhão auricular, e *pectus excavatum* . Todas as contraturas presentes eram rígidas. O paciente foi diagnosticado com camptodactilia de tipo III, e optou-se pelo tratamento conservador com ortetização em novembro de 2013. Não foi feita a goniometria inicial do paciente devido à dificuldade de execução, mas fez-se o registro fotográfico ( Fig. 1A-E ). As contraturas em flexão das articulações IFPs apresentavam mais de 90°. O uso de órteses estáticas inicialmente foi feito de forma ininterrupta, 8 e os retornos se deram a cada três meses, para acompanhamento e ajustes ou trocas de órteses.

**Fig. 1:** Características fenotípicas do paciente com Síndrome de Beals Heacht. Paciente com 2 meses ( **A** ), deformidades no punho, nas articulações metacarpofalangeanas e inferfalangeanas ( **B** ), anomalias no pavilhão auricular ( **C** ), *pectus excavatum* ( **D** ), e órtese de posicionamento com apoio dorsal ( **E** ).

O processo de ortetização teve seu início aos sete meses de idade. O objetivo inicial foi chegar à posição em neutro da flexão da articulação IFP, da flexão do punho, e da hiperextensão da articulação MCF, alcançada em fevereiro de 2015 ( Fig. 2 A-E ).

**Fig. 2:** Demonstração da evolução do tratamento conservador do paciente com camptodactilia. Início da correção ( **A** ), órteses de posicionamento com manutenção do apoio dorsal ( **B** ), órteses de uso noturno com polegares livres e placa de acetato-vinilo de etileno (EVA) na região dorsal de dedos ( **C** ), órteses de uso noturno em vista lateral ( **D** ), e extensão completa de dedos ( **E** ).

Após alcançar a posição em neutro, a órtese foi modificada, iniciando com uma leve flexão da articulação MCF e dispositivo de apoio dorsal seguindo a angulação da órtese, para exercer pressão nos dedos contra a órtese, mantendo assim a posição, além de auxiliar a flexão da articulação MCF. A partir de julho de 2015, já com 26 meses, as órteses passaram a ser confeccionadas com os polegares livres, as articulações MCF fletidas, e o punho em extensão ( Fig. 2, A-D ).

O uso das órteses passou a ser feito apenas no período noturno, em torno de 8 horas, a partir junho de 2016. Foi alcançada a correção da camptodactilia ( Fig. 2, E e Fig. 3 A-C ).

**Fig. 3:** Morfologia do membro superior após o tratamento conservador por 23 meses e meio. Posição supina com dedos em extensão ( **A** ), vista lateral ( **B** ), e dedos fletidos ( **C** ).

## Discussão

Neste caso de camptodactilia de tipo III rígida em criança com síndrome de Beals Heacht, a intervenção precoce 9 com órteses estáticas proporcionou desfecho satisfatório, com a regressão da deformidade e a obtenção do uso funcional das mãos. O uso de órteses deve ser a primeira escolha para o início do tratamento, inclusive nos casos sindrômicos. 9

A multiplicidade de estruturas envolvidas na camptodactilia que são responsáveis pelo desequilíbrio articular podem chegar a um equilíbrio com a remodelagem tecidual por meio de órteses. 9

A utilização de órteses para a remodelação do tecido musculoesquelético é uma opção de baixa complexidade; porém, é necessário um período de uso relativamente longo para que a remodelação ocorra. 10

O uso do dispositivo de apoio dorsal otimizou o alongamento das estruturas responsáveis pela contratura em flexão na articulação IFP.

O acompanhamento clínico do paciente em questão será mantido até o final do crescimento esquelético, para evitar possível recidiva de contratura de partes moles. 4 8

O envolvimento da família e sua colaboração foi um diferencial neste tratamento conservador. A intervenção é relativamente longa e exaustiva, mas é necessária. 10
